# A pilot study to evaluate the erythrocyte glycocalyx sensitivity to sodium as a marker for cellular salt sensitivity in hypertension

**DOI:** 10.1038/s41371-022-00683-z

**Published:** 2022-04-12

**Authors:** Ryan J. McNally, Franca Morselli, Bushra Farukh, Phil J. Chowienczyk, Luca Faconti

**Affiliations:** King’s College London, Department of Clinical Pharmacology, British Heart Foundation Centre, London, UK

**Keywords:** Hypertension, Risk factors

## Abstract

Supressed plasma renin in patients with primary hypertension is thought to be an indirect marker of sodium-induced volume expansion which is associated with more severe hypertension and hypertension-mediated organ damage. A novel test for erythrocyte glycocalyx sensitivity to sodium (eGCSS) has been proposed as a direct measure of sodium-induced damage on erythrocyte surfaces and a marker of sensitivity of the endothelium to salt in humans. Here we explore if eGCSS relates to plasma renin and other clinical and biochemical characteristics in a cohort of patients with primary hypertension. Hypertensive subjects (*n* = 85, 54% male) were characterised by blood biochemistry (including plasma renin/aldosterone), urine analysis for albumin-creatinine ratio (ACR), 24-h urine sodium/potassium excretion. eGCSS was measured using a commercially available kit. Correlations between eGCSS and clinical and biochemical characteristics were explored using Spearman’s correlation coefficient and characteristics compared across tertiles of eGCSS. eGCSS was inversely correlated with renin (*p* < 0.05), with renin 17.72 ± 18 µU/l in the highest tertile of eGCSS compared to 84.27 ± 146.5 µU/l in the lowest (*p* = 0.012). eGCSS was positively correlated with ACR (*p* < 0.01), with ACR 7.37 ± 15.29 vs. 1.25 ± 1.52 g/mol for the highest vs. lowest tertiles of eGCSS (*p* < 0.05). eGCSS was not correlated with other clinical characteristics or biochemical measures. These results suggests that sodium retention in hypertension characterised by a low-renin state is associated with cell membrane damage reflected by eGCSS. This may contribute to the hypertension-mediated organ damage and the excess mortality associated with sodium overload and “salt sensitivity”.

## Introduction

Hypertension is the leading preventable cause of premature death and disability worldwide [1]. Excess dietary salt intake is a major contributing factor to the pathogenesis of hypertension, this being associated with water retention, increase in systemic peripheral resistance, endothelial dysfunction and disruption of autonomic regulation of the cardiovascular system [2]. A proportion of hypertensive subjects, particularly those of African ancestry, exhibit a “salt-sensitive” phenotype [3] which differs in terms of the severity of hypertension [4], susceptibility to target organ damage [5–7] and response to treatment [8]. This is thought to be due to retention of sodium at the level of the renal tubules with damage to target tissues, including the endothelium [9, 10] It has been proposed that sodium overload damages the endothelial glycocalyx and diminishes the endothelial sodium buffer capacity [11] facilitating sodium deposition in various tissues [12, 13]. At the same time, sodium retention leads to an expansion of the extracellular fluid volume [14] thought to be responsible for a suppressive action on renin secretion [15] resulting in the characteristic ‘low-renin hypertension phenotype’ [16, 17].

**Fig. 1:**
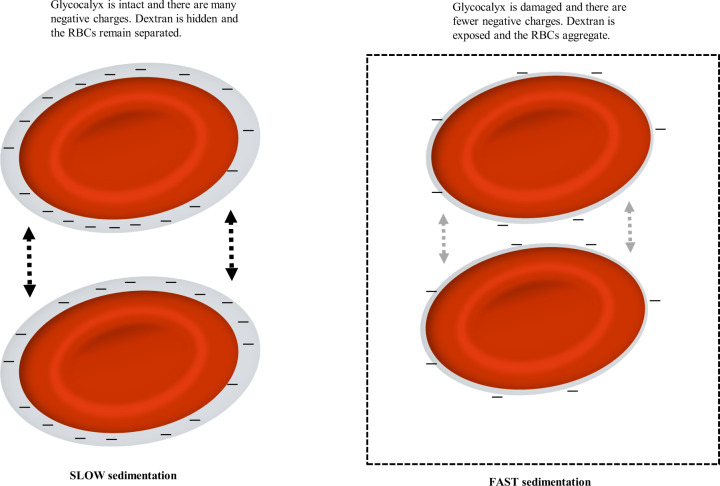
Schematic representation of the eGCSS and red blood cell (RBC) sedimentation. Figure adapted from Oberleithner et al. [19]. RBCs with an intact glycocalyx (left diagram) tend to remain separated but when the glycocalyx is damaged (right diagram), cells tend to aggregate.

No biomarker is currently available to measure salt sensitivity at a cellular level in humans [18] but recently it has been proposed that the effects of long-term sodium damage can be quantified by evaluating the erythrocyte glycocalyx sensitivity to sodium (eGCSS), a test previously referred to as a “salt blood test” [19]. Vascular endothelium is equipped with two salt-sensitive barriers in series, the endothelial glycocalyx [20] (where osmotically inactive sodium is bound) and the endothelial cell membrane that contains sodium channels [21]. The endothelial glycocalyx is a negatively charged mesh of membranous glycoproteins and proteoglycans, the majority of which are negatively charged heparan sulphate proteoglycans [11]. The glycocalyx of erythrocytes is thought to reflect the surface properties of endothelial cells [22] and the test has been proposed as a measure of the endothelial cell sensitivity to sodium in humans [23]. Both erythrocyte and endothelial glycocalyces selectively bind sodium and elevated concentrations of plasma sodium gradually destroy the charged surface layers of endothelium and red blood cells [24, 25]. When sodium buffering capacity of the glycocalyx is damaged by excessive sodium intake over time, this causes a reduction in the negatively charged heparan sulphate residues [26]. In fact, by using a mechanical nanosensor to measure the height and stiffness of the endothelial glycocalyx, it has been demonstrated that increased extracellular sodium concentration over 5 days led to a shrinkage and stiffening of the erythrocyte membrane and decrease of the heparan sulphate residues by 68% [11]. In turn, this influences the zeta potential which represents the magnitude of negative force that exists between red cells which in turn affects the velocity of red blood cells sedimentation (the variable measured by the eGCSS) (Fig. 1) [19].

For eGCSS, blood is exposed to a standard concentration of sodium [19, 23] that prevents haemolysis but maintains a sufficient moiety of free negative red blood cell surface charges, which is the critical variable for the sedimentation rate. In contrast to the erythrocyte sedimentation rate which is affected by inflammatory response and plasma proteins (such as fibrinogen), eGCSS is a more selective marker of the binding of sodium to the heparan sulphate residues on the erythrocyte surface [19].

The purpose of the present study, therefore, was to examine if eGCSS relates to plasma renin (used as a surrogate marker of sodium retention/volume expansion) and albumin/creatine ratio (as a sensitive measure of target organ damage) in a cohort of patients with primary hypertension. eGCSS was also performed in a small group of healthy volunteers to explore the correlation between results obtained with capillary and venous blood.

## Methods

### Subjects

Subjects with primary hypertension were recruited from the hypertension clinics at Guy’s and St Thomas’ Hospital NHS Foundation Trust, England, UK. Hypertension was diagnosed based on previous treatment and/or daytime ambulatory blood pressure (BP) (or home BP averaged over 7 days) of more than 135 mmHg systolic or more than 85 mmHg diastolic, according to current European Society of Hypertension guidelines [27]. Exclusion criteria included pregnancy, those in whom the clinical history or investigations suggested the presence of secondary hypertension, and patients with heart failure. Healthy volunteers were recruited by invitation. The study was approved by the local Research Ethics Committee in the UK and written informed consent was obtained from all patients.

### Erythrocyte glycocalyx sensitivity to sodium (eGCSS)

The protocol for the eGCSS has been described by Oberleithner et al. [23] with the original test performed on capillary blood. Venous blood was used for this study in hypertensive individuals with the correlation between the results obtained in capillary and venous blood examined in healthy volunteers (*n* = 10). eGCSS was measured in freshly drawn venous blood using a commercially available “salt blood test” (CARE Diagnostica, Voerde, Germany). Briefly, 50 µl of venous blood was mixed with a commercially available solution containing a pre-determined fix concentration of 90 mM Na^+^ (111 mM Na^+^ as a result of mixing 50uL of whole blood with 50 ul of 90 mM Na^+^) before being left to sediment for 60 min in a haematocrit tube [19, 23]. The length of the supernatant after 1 h was measured and the subsequent eGCSS calculated as a percentage relative to standard values of 21.4 and 26.1 mm for male and females, respectively [23]. These ‘standard’ values were used because the sodium buffer capacity of female blood is ~20% less than males because females have a lower haematocrit [23]. When this is accounted for values of eGCSS are similar in both sexes [23].

### Demographic and anthropometrics

Ethnicity was assigned according to “self-defined ethnicity” through questionnaires provided to the participants. Diabetes was diagnosed based on medical records or on glycosylated haemoglobin (HbA1c) at least 6.5% or 48 mmol/mol in individuals untreated by antidiabetic agents. Height was recorded using a stadiometer and body composition was measured using bioimpedance multi-frequency analysis with the TANITA Multi-Frequency Body Composition Analyser (MC-780MA, TANITA Corporation, Tokyo, Japan). Participants stood bare-footed on electrode platforms, with arms placed straight down by their sides whilst a small electrical current was passed through the body. Measurements were recorded in both kilograms and percentage. A tare weight of 1 kg was assumed for each patient.

### Hemodynamic measurements

Participants were asked to abstain from caffeine, alcohol and strenuous exercise for at least 24 h before the visit. Brachial blood pressure (BP) was measured by a trained observer after at least 15 min rest in a quiet temperature controlled vascular laboratory using an Omron HEM 705-CP semiautomatic oscillometric recorder (Omron Health Care, Tokyo, Japan). The average of two consecutive readings of systolic BP and diastolic BP and heart rate was used for the analysis.

### Biochemistry and urine analysis

Routine biochemistry and urine analysis were performed at ViaPath Laboratories, Guy’s and St Thomas’ Trust, London in line with standard procedures. Plasma biochemistry included full blood count, creatinine, electrolytes, HbA1c, cholesterol, plasma concentrations of renin and aldosterone which were measured from samples obtained after 15 min rest supine. Daily sodium and potassium excretion were estimated from an unsupervised 24-h urine collection. Participants were provided with a container and given careful instructions to collect all the urine produced over 24 h, making every effort to avoid incomplete collection. A spot urine sample was taken for analysis of albumin-creatinine ratio (ACR) to detect presence of microalbuminuria.

### Statistical analysis

Descriptive statistics are reported as mean ± standard deviation unless otherwise stated and as numbers and percentages for categorical variables. Correlations between eGCSS and clinical characteristics were examined using Spearman’s rank correlation coefficient. Differences in characteristics between tertiles of GCSS were analysed using one-way analysis of variance with post hoc LSD analysis used to assess the significance of differences between tertiles. Non-normally distributed variables were log-transformed prior to analysis. A *χ*^2^ test was used for categorical values. A *p* value < 0.05 was considered statistically significant and all tests were two-tailed. SPSS Statistics Version 25 (IBM Corporation, Armonk, New York, USA) was used for all statistical analysis. GraphPad Prism 8 (GraphPad Software Inc., La Jolla, California, USA) was used for graphical representation of data.

## Results

**Fig. 2:**
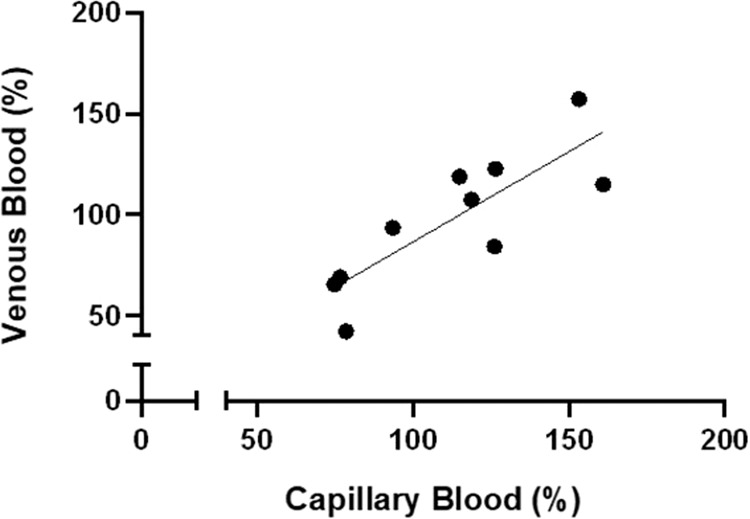
Correlation between eGCSS % measured with capillary vs. venous blood in *N* = 10 healthy volunteers.

In healthy volunteers there was a close correlation between the eGCSS measured in capillary blood and in venous blood (*R* = 0.83, *p* < 0.005, Fig. 2). Eighty-five subjects (54% male) with primary hypertension (aged 45.3 ± 12.2 years) were studied. Approximately half of the subjects were from non-Caucasian backgrounds, in line with the demographic of the area in south east London where the hospital is located [28]. Most of the participants (~75%) were treated with one or more antihypertensive medications, with the average number of treatments being 1.37 ± 1.21. Six subjects were diagnosed with type 2 diabetes.

**Fig. 3:**
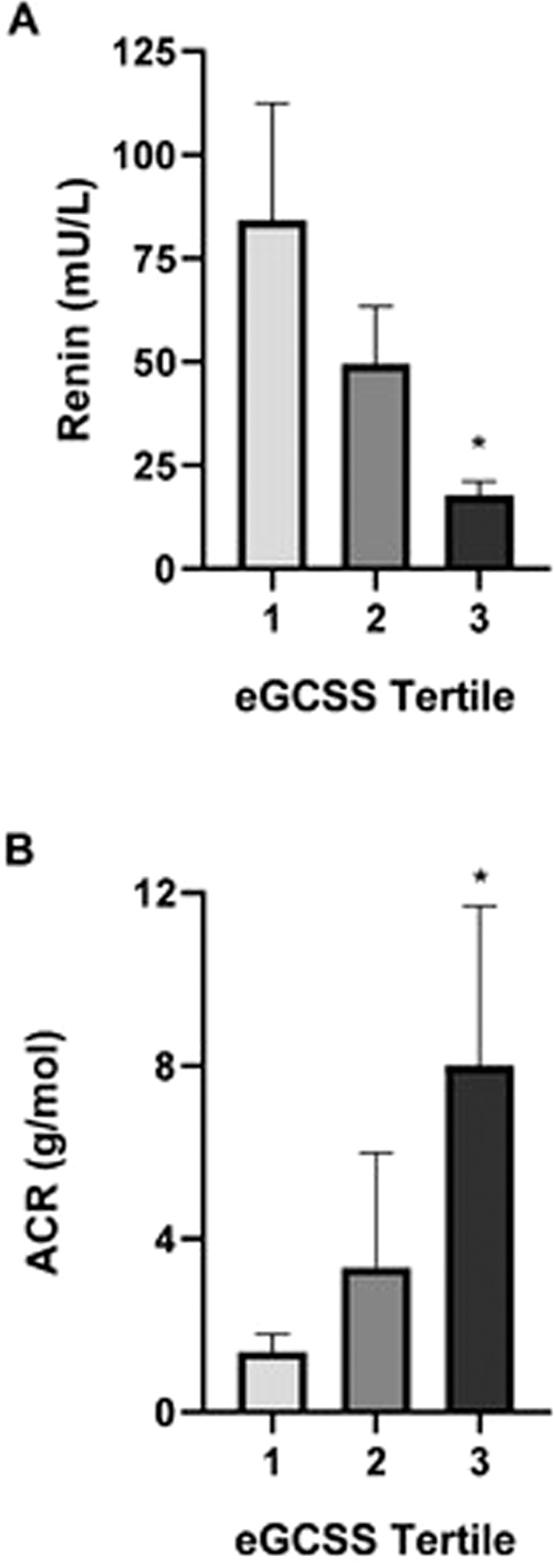
Stratification according to eGCSS tertile. **A** Renin, **B** albumin creatinine ratio (ACR). *p < 0.05 (tertile 3 vs. tertile 1).

Characteristics of hypertensive subjects stratified according to tertile of eGCSS are presented in Table 1. Those in the highest tertile had values of eGCSS in the range 144.85–221.96% which would be considered “salt-sensitive” according to previous results. The three groups were similar with respect to demographics and clinical characteristics (including age, gender, BP values, body composition, ethnicity and prevalence of diabetes) but renin was lower in those in the highest tertile of eGCSS compared to the lowest tertile (17.72 ± 18 µU/l vs. 84.27 ± 146.5 µU/l, *p* = 0.012, Fig. 3). Similar results were seen when patients (8/85) treated with beta-blockers were excluded from the analysis. Differences in haematocrit were also considered and when eGCSS was adjusted for an average HCT of 41.55, the eGCSS values were 87.55%, 128.73% and 173.68% for each tertile respectively (*p* < 0.001). ACR was also higher in the highest compared to the lowest tertile of eGCSS (7.37 ± 15.29 g/mol vs. 1.25 ± 1.52 g/mol, *p* < 0.05, Fig. 3). There was no difference in other biochemistry including plasma sodium, potassium, creatinine and urinary excretion of sodium and potassium (Table 1). In the entire population, there was a negative correlation between eGCSS and plasma renin (*R* = 0.26, *p* = 0.016) and a positive correlation with ACR (*R* = 0.37, *p* = 0.003) (see Supplementary Fig. 1). eGCSS was greater in hypertensive subjects compared to values in heathy normotensive volunteers (122.75 ± 39.8%, vs. 97.48 ± 33.56%, *p* < 0.05).

**Table 1:** Demographics, anthropometric, hemodynamic and biochemical characteristics of the study population (*n* = 85) stratified by tertile of eGCSS.

	eGCSS Tertile			*p* value
	86.27 ± 13.02 (53.7–107.3)	127.89 ± 10.41 (107.5–142.7)	174.35 ± 21 (144.9–222)	
Demographics and anthropometrics				
Age (years)	45 ± 12	43 ± 11	48 ± 13	0.264
Male (%)	51.9	58.6	51.7	0.835
Ethnicity (white %)	48.1	65.5	37.9	0.310
BMI (kg/m^2^)	29.33 ± 5.82	29.20 ± 4.22	29 ± 5.17	0.977
Body water (%)	52.84 ± 6.04	53.07 ± 6.77	53.30 ± 7.78	0.982
Body fat (%)	28.64 ± 7.26	27.83 ± 8.23	27.28 ± 9.90	0.901
Pharmacological treatment (%)	77.7	58.6	82.1	0.235
Average no. of drugs	1.33	1.26	1.5	0.756
Haemodynamics				
Systolic BP (mmHg)	142 ± 15	140 ± 15	144 ± 17	0.656
Diastolic BP (mmHg)	92 ± 10	87 ± 9	89 ± 13	0.288
Heart rate (bpm)	76 ± 80	76 ± 19	76 ± 12	>0.99
Biochemistry				
Creatinine (µmol/l)	78 ± 17	75 ± 13	85 ± 26	0.167
Sodium (mmol/l)	140 ± 1.67	140 ± 1.70	141 ± 2.14	0.668
Potassium (mmol/l)	4.23 ± 0.41	4.21 ± 0.48	4.29 ± 0.35	0.790
24-h sodium (mmol/24 h)	126 ± 73	144 ± 98	164 ± 89	0.301
24-h potassium (mmol/24 h)	59.70 ± 13.79	78.23 ± 21.24	76.27 ± 32.03	0.146
Volume collected (ml)	1823 ± 523	2000 ± 640	2010 ± 689	0.658
HbA1c (mmol/mol)	45.24 ± 25	40.95 ± 12	39.85 ± 5.32	0.562
Total cholesterol (mmol/l)	4.66 ± 0.84	5.04 ± 0.98	4.69 ± 1.01	0.375
Renin (µU/l)	84 ± 147	50 ± 75	18 ± 18	**0.033**
Aldosterone (pmol/l)	399 ± 219	522 ± 662	368 ± 188	0.378
Albumin/creatinine ratio (g/mol)	1.25 ± 1.52	2.86 ± 9.02	7.37 ± 15.29	**0.048**

Bold values indicate statistical significance *p* < 0.05.

*p* values represent one-way ANOVA.

## Discussion

Our pilot data show that hypertensive subjects with higher eGCSS exhibit a lower plasma concentration of renin and higher urinary ACR, despite no difference in other cardiovascular risk factors such as level of BP or body composition. This is consistent with eGCSS being a marker of sodium-induced damage at cellular level with associated damage to microvascular function reflected by increased urinary albumin excretion. eGCSS in healthy volunteers was lower than in subjects with hypertension, confirming previous published data [29], and results obtained from capillary and venous blood correlated well suggesting the test can conveniently be applied to venous blood that is collected for routine biochemistry.

Salt intake is well recognised to contribute to hypertension and related target organ damage [30–35] but response to salt ingestion varies within and across populations. One measure of “salt sensitivity” is the degree to which BP increases in response to increased salt consumption [36], usually defined as a change in BP of 5–10% or at least 5 mmHg in response to a change in dietary sodium chloride intake [37]. Hypertension mediated target organ damage in salt-sensitive individuals resembles that in animal models, with a predominance of left ventricular hypertrophy, renal damage, and stroke [38, 39]. The BP response to salt is a predictor of severity of hypertension-mediated organ damage [5] and a risk factor for cardiovascular mortality and morbidity, independent of and as powerful as the level of BP itself [36]. Results of the present pilot study might support the concept that eGCSS reflect the damage to endothelial cell membranes by sodium [22]; however larger studies with more carefully characterised patients will be required to confirm this.

This pilot study is subject to several limitations. First a relatively small sample size limits the ability to detect associations with characteristics such as 24-h urinary sodium which exhibited relatively large mean differences between eGCSS tertiles that did not reach statistical significance. Most of the patients were currently taking antihypertensive medication at the time of measurement and diet/salt intake was not controlled. Plasma renin was used as marker of sodium-induced volume expansion and is influenced by many factors other than salt sensitivity (including drugs, plasma levels of aldosterone and sodium intake) [40]. Further studies in carefully selected untreated groups of patients with standardisation of dietary sodium intake will be required to clarify which of these factors is most strongly associated with eGCSS. Many studies show that cellular damage by sodium is amplified by higher concentrations of aldosterone, and it would be particularly interesting to determine if eGCSS is elevated in patients with primary aldosteronism.

Nevertheless, the study offers proof-of-concept that eGCSS is a potentially useful marker of sodium sensitivity at cellular level. Further work is required to distinguish whether eGCSS is a more direct measure of cellular sensitivity to the damage induced by sodium than renin and if its correlation to hypertension mediated target organ damage is superior to that of renin. Finally, it needs to be clarified if and to what degree eGCSS is influenced by nonspecific damage to the endothelium and its correlation with markers of inflammation.

In conclusion, the present result suggests that low-renin state is associated with higher eGCSS which might reflect the damage of the cell membrane induced by sodium. Further studies to examine the utility of eGCSS as a biomarker of target organ damage, BP response to salt and pharmacological treatment are warranted.

### Summary table

#### What is known about this topic

- No biomarker is currently available to measure salt sensitivity at a cellular level in humans.
- Recently it has been proposed that the effects of long-term sodium damage can be quantified by evaluating the erythrocyte glycocalyx sensitivity to sodium, a test previously referred to as a “salt blood test”.

#### What this study adds

- Erythrocyte glycocalyx sensitivity to sodium is a potentially useful marker of sodium sensitivity at cellular level.
- This study suggests that sodium retention in hypertension characterised by a low-renin state is associated with cell membrane damage which may contribute to the hypertension-mediated organ damage.

## Supplementary information

Supplementary Material
