# Supplementary material for: A pilot study to evaluate the erythrocyte glycocalyx sensitivity to sodium as a marker for cellular salt sensitivity in hypertension
Source: J Hum Hypertens. 2022 Apr 12;37(4):286–91. doi: 10.1038/s41371-022-00683-z (PMC10063440; doi:10.1038/s41371-022-00683-z)
Supplement: Supplementary file 1 — Supplementary Material [file 41371_2022_683_MOESM1_ESM.docx]

**Supplementary Material**

**Figure 1:** Correlation between eGCSS and plasma renin and ACR
